# Scientific education in German medical schools: nationwide cross-sectional study reveals student needs and gaps

**DOI:** 10.1186/s12909-026-09311-7

**Published:** 2026-05-02

**Authors:** Maximilian Vogt, Mark Enrik Geissler, Sebastian Gerdes, Jean-Paul Bereuter, Nadja Schuchardt, Rona Berit Geissler, Ingmar Glauche, Ingo Roeder, Andreas Deußen, Lydia Günther

**Affiliations:** 1 Division of Medical Biology, Department of Psychiatry and Psychotherapy, Faculty of Medicine and University Hospital Carl Gustav Carus, TUD Dresden University of Technology, Fetscherstraße 74, Dresden, 01307 Germany; 2 Department of Neurosurgery, University Medicine Greifswald, Ferdinand-Sauerbruch-Straße, Greifswald, 17475 Germany; 3 Medical Clinic III, Gastroenterology, Metabolic Diseases and Intensive Care, University Hospital RWTH Aachen, Aachen, Germany; 4 Institute for Medical Informatics and Biometry, Faculty of Medicine Carl Gustav Carus, TUD Dresden University of Technology, Dresden, Germany; 5 Department of Visceral, Thoracic and Vascular Surgery, Faculty of Medicine and University Hospital Carl Gustav Carus, TUD Dresden University of Technology, Fetscherstraße 74, Dresden, 01307 Germany; 6 Department of Physiology, Medical Faculty, Carl Gustav Carus, Technische Universität Dresden, Dresden, Germany

**Keywords:** Scientific training, Medical education, Competency assessment, German medical students

## Abstract

**Introduction:**

Medical graduates must integrate new scientific findings into clinical practice requiring strong scientific training. In Germany, scientific education in medical curricula is often undervalued, necessitating curricular changes. This study evaluates medical students' current scientific training, perceived and objective knowledge, and their preferences for curricular organisation.

**Methods:**

A nationwide cross-sectional study was conducted using an online survey distributed to medical students across 45 German medical schools. The survey, conducted from March to May 2023, covered scientific education aspects including self-assessment of scientific skills and an optional 25-item knowledge competency test. Data were collected from 3005 students, with 1319 completing the full competency test.

**Results:**

Only 53.8% of students were aware of their scientific curriculum, and over 60% reported no evaluation of their scientific skills at their universities. In their final year, 52.7% felt competent in literature search, and 44.1% in scientific writing. However, only 19.9% felt competent in study design, 25.7% in developing research projects, and 19.8% in applying findings to patient care. The average competency test score for final-year students was 16 out of 25, with notable deficiencies in empirics and practical applications. At least 62.2% of students expressed a desire for more scientific training, and 71.3% favoured mandatory scientific courses.

**Conclusion:**

German medical students are dissatisfied with their current scientific education with 75% expressing dissatisfaction. They feel unprepared to apply scientific knowledge in clinical settings. The study highlights the need for urgent curricular reforms to enhance practical scientific training and better prepare future physicians for modern medical practice and research.

**Supplementary Information:**

The online version contains supplementary material available at 10.1186/s12909-026-09311-7.

## Introduction

**Fig. 1:**
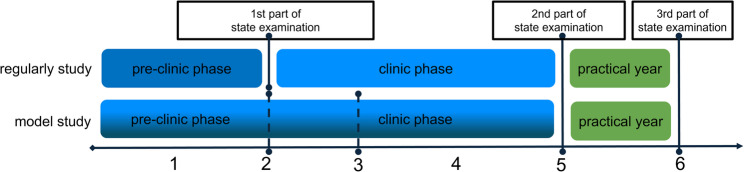
Overview of German medical curricula. The general structure of the German medical degree programme is shown. A formal distinction is made between regular and model programmes. Model curricula are often organised as integrated curricula, but still variations exist. The 1 st part of the state examination is not mandatory in model curricula, but is sometimes implemented in different ways (dotted lines). The practical year is obligatory and begins after five years of study

The recent century has seen an unprecedented rise in medical research and knowledge generation leading to an exponential growth of scientific literature and increasingly complex data [1, 2]. Implementing and validating these advancements leads to continuous growth in clinical research and guidelines [3] as well as adaptation in already densely packed curricula [4]. Physicians need to master these challenges by closely following scientific developments and being prepared for lifelong learning essential for both clinical and research settings [5]. Only then, they can evaluate and implement up-to-date evidence into their clinical practice with patients and, vice versa, actively translate clinical challenges, in diagnostics, treatment and beyond, into translational research. Particularly clinician scientists face significant challenges [6, 7]. For decades their numerical decline has been addressed [8], and the strengthened inclusion of scientific training is of utmost importance [9]. The implementation of fundamental scientific knowledge varies by country and medical school system [10]. In the United States a bachelor’s degree is required, and medical education includes basic science and clinical exposure over three to four years, with Doctor of Medicine (MD)/Doctor of Philosophy (PhD) programs providing protected research time [11, 12]. In the United Kingdom, medical school combines five-year bachelor’s and consecutive master’s in medicine and surgery, with common MD/PhD options [13]. The German system offers six years of training without requiring a bachelor’s or master’s degree, focusing on basic science followed by clinical education [12] (Fig. 1). In the 6th year, students go through clinical rotations. Some medical schools include clinical training already earlier (model study). The MD title acquired through a medical thesis is obtained by around two-thirds of students, with additional PhDs being less common [14, 15]. Scientific training in medical curricula is essential [16], and there has been a call for more scientific education in Germany [5, 17–20]. Graduate profiles such as the German National competency-based learning outcomes catalogue (NKLM) [21] acknowledge this need, but integration varies significantly between medical schools, with few universities introducing mandatory research projects [22]. Furthermore, a draft of the new German medical licensing regulations encourages the incorporation of scientific training, which is to be completed with a compulsory 12-week research project [23]. This ongoing discussion emphasises the need to evaluate the impact of recent curricular interventions on students’ scientific awareness, preparedness, and competency. Our nationwide cross-sectional study aims to:

- Describe the current state of scientific training among medical students from a students’ perspective and to analyse their current scientific competencies.
- Identify factors that influence self- and objective-assessment scores.
- Explore the students' opinions on current scientific training and their preferences for curricular interventions.

## Methods

### Survey design

This cross-sectional study was performed as an online survey using LimeSurvey 3. We designed the survey in an iterative collaborative process based on Vogt et al., 2024 [24]. All questions were evaluated by a group of medical students, who did not take part in the survey, to assess the amount of time required and to remove ambiguity. The questionnaire (Suppl. 1) comprised eight different topics. We requested demographic details, status of medical thesis, scientific content in the curriculum, relevance of scientific competencies in professional practice, and self-assessment of scientific skills by a verbal rating scale. This self-assessment was based on a 12-item questionnaire. The scales of the questionnaire defined different competence levels from no competence to independent performance. Additionally, a 25-item multiple-choice competency test was offered. The test covered 5 different science-related topics (theory, ethics, study design, empirics, practice) with 5 questions each. Completing the survey required approximately 30 min. Questions were developed according to the competencies and learning objectives defined in the German NKLM, covering the previously mentioned areas for scientific education [21].

### Distribution and data acquisition

The survey was distributed to German medical students enrolled at 45 universities with different curricular structure (Fig. 1) from March to May 2023 in cooperation with the Federal Association of Medical Students. Survey participation was incentivized through a voucher raffle.

### Participants and data protection

The study including the experimental protocol and the terms of realization has been approved by the Ethical committee at the Technical University of Dresden (SR-EK-152032023) as well as by the data protection officer. Informed consent was used. Human Ethics and Consent to Participate declarations: not applicable.

The participation was voluntary. The data was collected anonymously and stored on servers of the TU Dresden. Clinical trial number: not applicable.

### Data treatment and analysis

Python 3.11.8 was used for data analysis within the Visual Studio Code 1.87.1 environment. The detailed methods and data from complete questionnaires are available (Suppl. 2, 3). Previous education was categorized into healthcare-related studies (e.g. psychology), healthcare-related education (e.g. physiotherapy), and non-healthcare-related education (e.g. economics). Parameters such as age and high school grade were analysed descriptively (means and standard deviations). Verbal rating scale questions were analysed using percentages. Self-assessment scores were converted into a competency-oriented scale from 0 (can do nothing), 1 (can name facts), 2 (can explain), 3 (can perform under supervision) to 4 (can perform independently). Hence, the twelve items of the subjective assessment result in a score from 0 to 48. The competence test with 25 questions (Suppl. 1) resulted in a score ranging from 0 to 25, and secondly the questions were divided into 5 equally sized categories: theory (e.g. inductive/deductive), ethics (e.g. ethics committee), study design (e.g. subgroups), empirics (e.g. sensitivity), and practice (e.g. bias, citation). Internal consistency was assessed using Cronbach’s alpha for both, the competence test and the self-assessment. Multiple linear regressions were used to analyse the influence of the curriculum on the assessment scores, whereby these scores were considered in relative terms (in % of specific assessment score). Controls for potential confounders like high school Grade Point Average (GPA) and career aspirations were included, excluding effects with a variance inflation factor over 5 to prevent multicollinearity. We categorized independent variables into quantiles (e.g. high school GPA, relevance). Only fully completed questionnaires (all 25 optional multiple-choice questions answered) were analysed, whereby subgroups that were too small were excluded (“others” for career prospects, sex, high school grade). We used the Student’s t-test to test for difference of means between two groups. The level of significance was set to alpha = 0.05. In our analysis, box plots depict the interquartile range, with whiskers extending to 1.5 times the interquartile range and the median distinctly marked inside the box. Bar plots are shown with whiskers extending to two times the standard deviation.

### Funding

Open Access funding enabled and organized by Projekt DEAL. The project was funded by FOSTER (012–2023 and A4-2022), an initiative by the Federal Ministry of Education and Research (BMBF) and the state of Saxony under the Excellence Strategy of the Federal Government and the State Government. The project was further funded by a MeDDrive Teaching Fond (65577) of the MFD.

## Results

### Sample characteristics

Nationwide 4133 medical students from 45 medical schools (n/regular study = 32; n/model study = 13) participated. 3005 completed the questionnaire (Table 1). Females predominated (69.6%). A majority lacked previous healthcare-related experience (68.7%), in contrast to about 20.7% with prior training. The academic MD degree was a common goal for 87.0% of the participants, whereas 11.2% were undecided, and 1.8% did not aim to perform a medical thesis. The mean high school GPA was similar across all academic years (AY) (1.47 SD 0.50). The mean high school GPA was 1.47 (SD 0.50). Participants’ age commenced at 20.5 years (SD 2.2) and increased by approximately one year per AY. For details regarding the different faculties, see Suppl. 4.

**Table 1:** Survey sample distribution and demographic data per academic year (AY)

	1. AY	2. AY	3. AY	4. AY	5. AY	≥ 6. AY
participating students, *n*	657	610	619	518	379	222
Sex (n, %)						
male	199 (30.2 %)	153 (25.0 %)	177 (28.6 %)	162 (31.3 %)	110 (29.0 %)	92 (42.4 %)
female	451 (68.6%)	454 (74.4%)	437 (70.6%)	354 (68.4%)	269 (71.0%)	129 (58.1%)
diverse	4 (0.5%)	2 (0.2%)	3 (0.3%)	1 (0.2%)	0 (0%)	1 (0.4%)
Medical thesis (n, %)						
strives for it	547 (83.3%)	477 (78.1%)	453 (73.2%)	201 (38.8%)	47 (12.4%)	26 (11.7%)
not planned	5 (0.76%)	12 (2.0%)	9 (1.50%)	11 (2.1%)	10 (2.6%)	5 (2.3%)
don’t know	103 (15.7%)	112 (18.4%)	68 (11.0%)	27 (5.2%)	18 (4.7%)	9 (4.1%)
started	2 (0.3%)	9 (1.5%)	85 (13.7%)	275 (53.1%)	290 (76.5%)	163 (73.4%)
cancelled	0 (0%)	0 (0%)	0 (0%)	3 (0.6%)	8 (2.1%)	6 (2.7%)
completed	0 (0%)	0 (0%)	4 (0.6%)	1 (0.2%)	6 (1.5%)	13 (5.9%)
others	0 (0%)	0 (0%)	0 (0%)	0 (0%)	0 (0%)	0 (0.0%)
Healthcare-related educational background (n, %)						
none	485 (73.8%)	381 (62.5%)	412 (66.6%)	366 (70.7%)	269 (71.9%)	152 (68.5%)
Healthcare- related education	112 (17.0%)	152 (24.9%)	142 (22.9%)	108 (20.8%)	70 (18.4%)	41 (18.5%)
Healthcare- related study	44 (6.6%)	45 (7.4%)	45 (7.3%)	34 (6.5%)	23 (6.0%)	18 (8.1%)
Non-Healthcare related education and/or study	16 (2.4%)	32 (5.1%)	20 (3.2%)	10 (1.9%)	17 (4.4%)	11 (5.0%)

### Description of the current scientific training from a students’ perspective

A substantial portion of students were unable to specify how scientific training is embedded in their curriculum even in the late AY (1st AY: 61.0%, *n* = 657 vs. > 6th AY: 34.2%, *n* = 222, data not shown). Looking at the students who knew what kind of curriculum they have, separate courses spread across the entire curriculum was the most frequently mentioned course type (40.0%) in modal value, being most common in 23 locations. A teaching course en bloc was the second most frequently mentioned course type (20.2%) but was the modal value at only 8 locations. Science education integrated into other courses (18.3%) was the modal value at 7 faculties. Optional integration, clinical or preclinical electives were less prevalent, with 10.5% and 8.9%, respectively. Less than 1%) reported a research semester (~ 6 months) as part of their curriculum. Focusing on the students in the > 6 AY (*n* = 222), the majority reported the absence of a dedicated examination for science education (73.4%), while only 18.9% confirmed the existence and 7.7% were not able to answer. Regarding practical experiences for scientific training, 60.8% of students in the > 6 AY reported none, whereas 27.4% acknowledged its availability at their faculty (11.7% stated no statement possible). The duration of these practical training opportunities varied from on average two weeks (6 faculties), with a maximum of 13 weeks as a research semester (2 faculties).

### Scientific competencies rated by self-assessment and through competency test

**Fig. 2:**
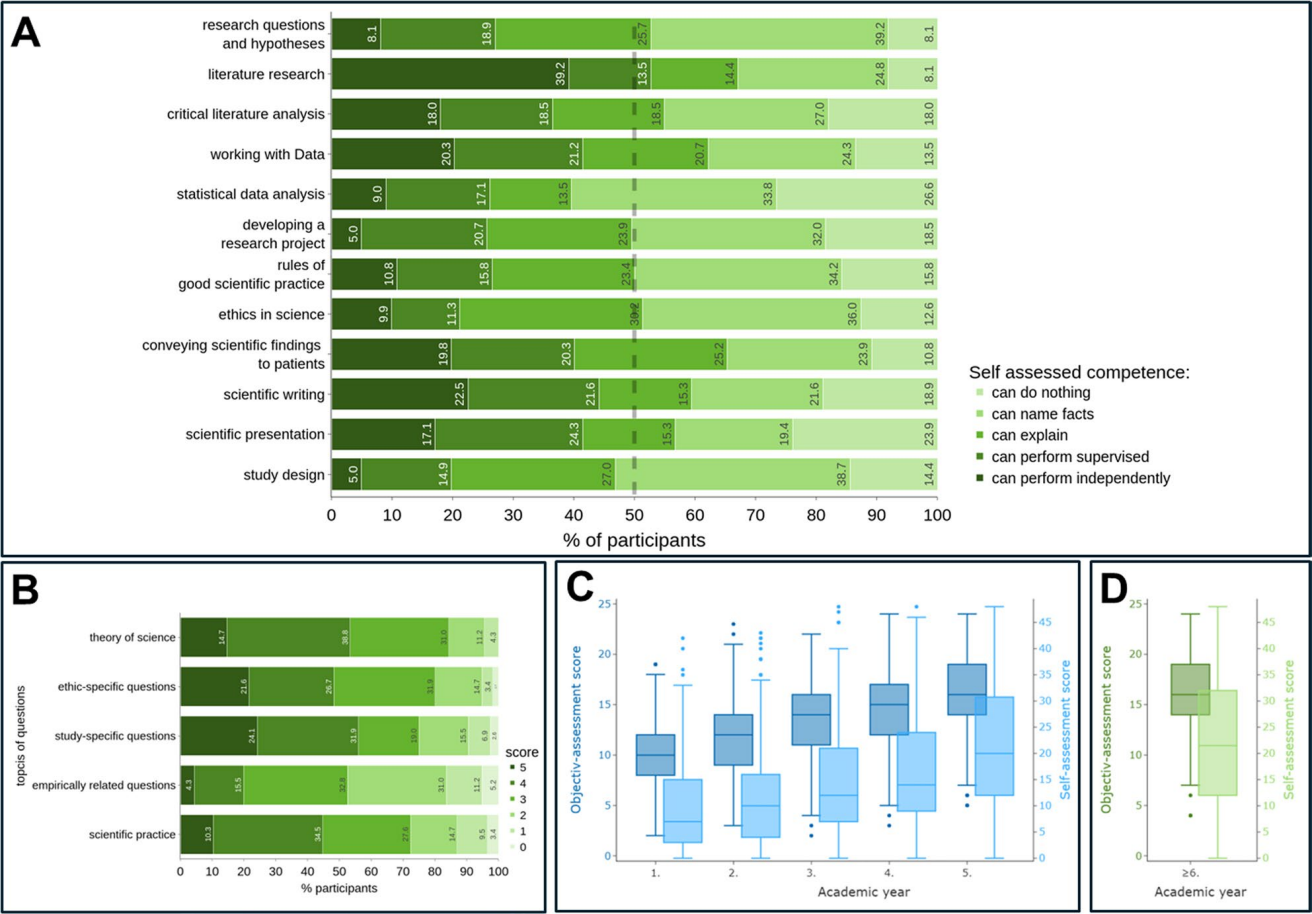
Self-assessed competence and results of the competency test. **A** Self-assessment by medical students in the ≥ 6th AY (*n* = 222) is plotted horizontally by stacked bar charts. The different competence levels are color-coded from low (light green) to high (dark green). The 50% mark is denoted by a dashed line. **B** The outcomes of the objective-assessment are shown for the ≥6th AY (*n* = 116) as horizontal stacked bar charts. It illustrates the distribution of scores ranging from 0 (light green) to 5 points (dark green) per question category along the y-axis. **C **The box plots show the results of the objective-assessment (maximum 25 points, n1stAY = 241, n2ndAY = 255, n3rdAY = 288, n4thAY = 242, n5thAY = 175) on the left y-axis (dark blue) and the results of the subjective assessment (maximum 48 points, n1stAY = 647, n2ndAY = 610, n3rdAY = 619, n4thAY = 518, n5thAY = 379) on the right y-axis (light blue) over the 1 st − 5th AY. **D** The box plots show the results of the objective assessment (maximum 25 points, *n* = 116) on the left y-axis (dark green) and the results of the subjective assessment (maximum 48 points, *n* = 222) on the right y-axis (light green) for the 6th AY

The students were asked to self-assess their current scientific competences (Cronbach’s alpha = 0.94, *n* = 3005). Among the students in their final year (≥ 6th AY, *n* = 222), more than 40% rated themselves as competent in performing literature research and its critical analysis, working with data, scientific writing and presentation as well as conveying scientific findings to patients. In contrast, the self-assessed competence of students at the performance level (under supervision or independently) regarding basic requirements when doing research i.e. generating hypotheses and research questions, developing and conducting research projects, rules of good scientific practice as well as statistical analyses, is developed only by around one in four students. Only around 20% assessed themselves as capable to perform study design and evaluate ethics in science independently or under supervision (Fig. 2A). This situation was quite similar in the lower academic years (data not shown).

The students also had the opportunity to complete a 25-item competency test (Cronbach’s alpha = 0.733, *n* = 1319). Among the students in their final year (> 6th AY, *n* = 116) the average score reached was 16 (SD 4.1). Low performance could be observed regarding the topic’s empirics and practice (Fig. 2B). An increase of self- and objective-assessment scores was notable with study progress. The highest scores were reached in the 5th AY but did not further increase in the final 6th AY (Fig. 2C and D). Improvement over the years was particularly marked in questions regarding study design (1st AY mean = 1.9 points, *n* = 240 vs. 6th AY mean = 3.4, *n* = 116, *p* < 0.001, Suppl.6), whereas less advancement was seen in questions related to empirics (1st AY mean = 1.6 points, *n* = 240 vs. 6th AY mean = 2.5, *n* = 116, *p* < 0.001, data not shown). Self-assessment and competency test scores showed a weak positive correlation (Spearman ρ = 0.33, *p* < 0.001).

### Analysis of factors that impact scientific competencies

Multiple linear regressions were used to investigate the associations between different approaches to integrate scientific education in curricula, self-assessment, and the competency test (Table 2). The type of curricular integration of scientific education was significantly associated with both self-assessment (F-test, *p* < 0.001) and competency test scores (F-test, *p* < 0.001). Students unable to identify the format of scientific education in their curriculum scored lowest in both measures. Study progress was among the strongest predictors - compared to preclinical students, those in late clinical years scored substantially higher in both scores. Previous healthcare-related studies were associated with significantly higher scores. A good high school GPA (0.7–1.2) was more likely to be associated with a higher competency test score, whereas a high relevance score (35–45 of maximum 45 points) for scientific skills in professional practice was more strongly associated with self-assessment than with competency test scores. Performing a doctoral thesis was associated with a strong and significant increase in self-assessment scores, but not with a significant increase in competency scores. Career aspirations showed no significant associations with either score.

**Table 2:** Predictors of objective assessment and self-assessment scores

		Objective Assessment						Self-Assessment					
		B	β	SE	CI lower	CI upper	*p*	B	β	SE	CI lower	CI upper	*p*
Intercept		34.81		2.17	30.56	39.06	< 0.001	13.83		3.02	7.92	19.75	< 0.001
High school GPA	*Bad (reference)*												
	Mid	1.61	0.044	1.14	−0.63	3.86	0.158	1.03	0.021	1.63	−2.17	4.23	0.527
	Best	5.46	0.158	1.21	3.08	7.84	< 0.001	1.39	0.030	1.74	−2.03	4.81	0.424
Academic year	*Preclinic (1–2 AY) (reference)*												
	Early clinic (3–4 AY	9.90	0.286	1.02	7.91	11.89	< 0.001	2.44	0.052	1.50	−0.50	5.38	0.103
	Late clinic (5–6 AY)	15.84	0.378	1.50	12.91	18.77	< 0.001	8.19	0.145	2.20	3.87	12.50	< 0.001
Advanced medical background	*None (reference)*												
	Healthcare-related education	2.46	0.059	1.22	0.06	4.86	0.044	−0.85	−0.015	1.74	−4.27	2.57	0.626
	Healthcare-related study	7.96	0.118	1.69	4.63	11.28	< 0.001	12.36	0.136	2.41	7.63	17.08	< 0.001
	Non-healthcare education/study	5.78	0.067	2.11	1.64	9.92	0.006	5.93	0.051	3.01	0.02	11.84	0.049
Career aspiration	*General practitioner (reference)*												
	Employed doctor in hospital	−0.11	−0.003	1.58	−3.22	2.99	0.942	−1.57	−0.030	2.25	−5.99	2.84	0.485
	Employed doctor at university hospital	2.05	0.054	1.61	−1.11	5.21	0.203	−1.38	−0.027	2.29	−5.88	3.12	0.547
	Specialist in private practice	−0.34	−0.010	1.51	−3.31	2.62	0.820	−3.12	−0.065	2.15	−7.33	1.09	0.147
Medical doctorate	*Undecided (reference)*												
	Strives for it	−2.23	−0.065	1.34	−4.85	0.40	0.097	3.84	0.083	1.91	0.09	7.58	0.045
	Not planned	−6.94	−0.055	3.31	−13.44	−0.44	0.036	1.30	0.008	4.72	−7.97	10.57	0.784
	Started	1.20	0.032	1.65	−2.05	4.44	0.470	14.49	0.289	2.32	9.94	19.05	< 0.001
Curriculum	*Unable to answer (reference)*												
	Separate course spread over study	5.34	0.132	1.08	3.22	7.47	< 0.001	6.56	0.120	1.54	3.53	9.58	< 0.001
	Block course	2.90	0.055	1.37	0.22	5.58	0.034	5.28	0.074	1.95	1.46	9.10	0.007
	Integration into other courses	7.38	0.136	1.41	4.60	10.15	< 0.001	5.49	0.075	2.02	1.52	9.46	0.007
	Optional course	5.60	0.104	1.42	2.82	8.39	< 0.001	1.75	0.024	2.03	−2.23	5.73	0.389
Self-assessment score	*Low (reference)*												
	Low-mid	3.60	0.086	1.26	1.12	6.08	0.004						
	Mid	2.95	0.070	1.29	0.42	5.49	0.023						
	Mid-high	1.49	0.035	1.34	−1.14	4.13	0.266						
	High	7.42	0.166	1.46	4.56	10.28	< 0.001						
Objective assessment score	*Low (reference)*												
	Mid							0.94	0.019	1.47	−1.95	3.83	0.524
	High							7.64	0.155	1.69	4.33	10.95	< 0.001
Relevance score	*Low (reference)*												
	Mid	2.25	0.060	1.02	0.24	4.26	0.028	1.32	0.026	1.46	−1.54	4.18	0.365
	High	2.29	0.063	1.03	0.26	4.32	0.027	6.62	0.134	1.47	3.75	9.50	< 0.001

Multiple linear regression analysis of factors associated with objective assessment and self-assessment scores (*n* = 1194). Model fit for Objective assessment R² = 0.330, adj. R² = 0.317, F(23, 1170) = 25.04, *p* < 0.001; Model fit for Self-assessment R² = 0.254, adj. R² = 0.241, F(21, 1172) = 19.03, *p* < 0.001

### Students opinion on current curricular scientific training

**Fig. 3:**
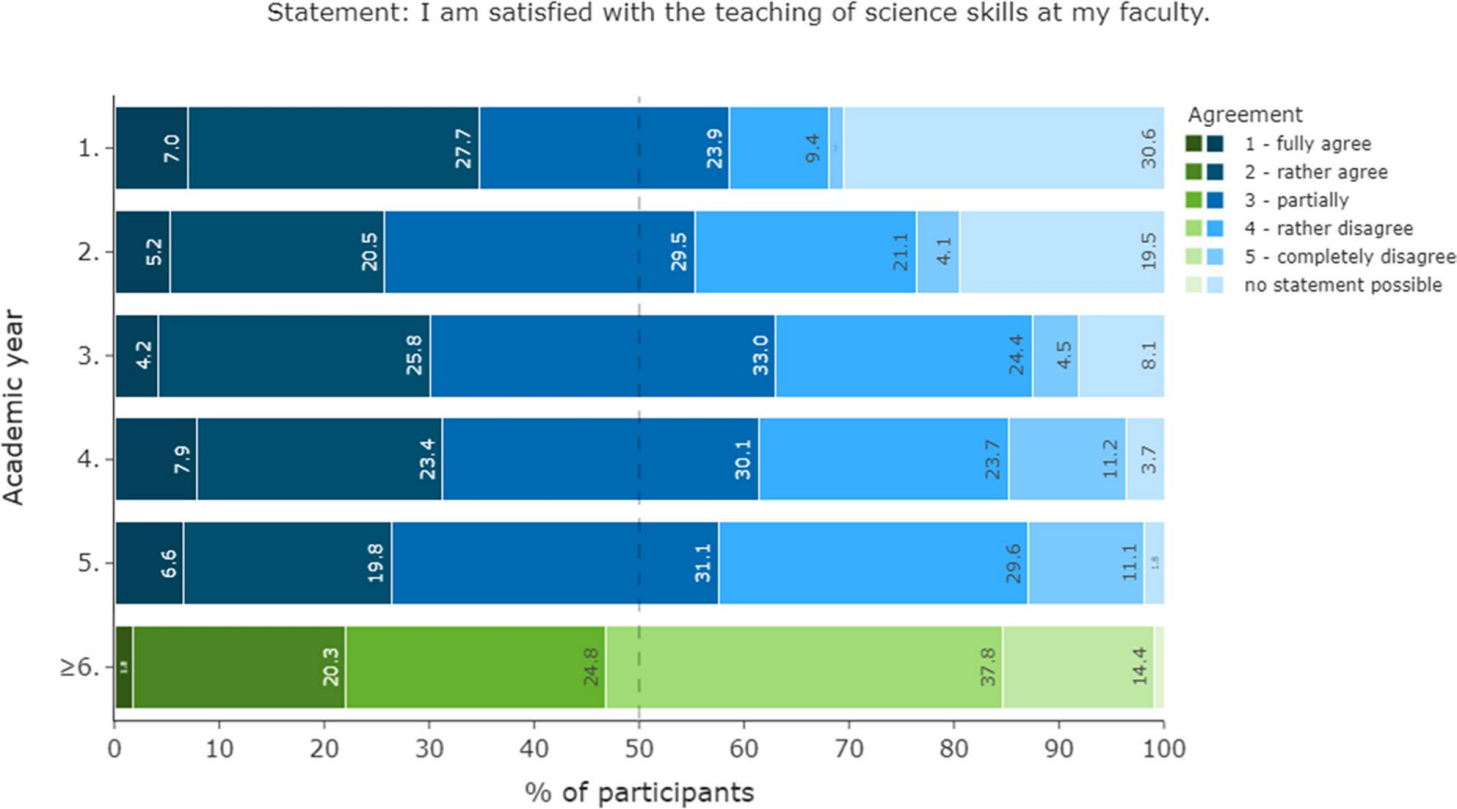
Student satisfaction with their scientific education. The responses are presented in horizontally stacked bar charts (*n* = 3005). The 50% mark is denoted by a dashed line. The stronger the agreement to the statement the darker the colour. The y-axis shows the different AYs. The first five AYs are shown in blue, while the graduates are shown in green colour grades

Students’ satisfaction decreased over AYs. Around 53% of the students in their final year of studies were dissatisfied and further 25% were only partially satisfied with their scientific education (Fig. 3, Suppl. 7).

### The students’ preferences for scientific training and curricular interventions

**Fig. 4:**
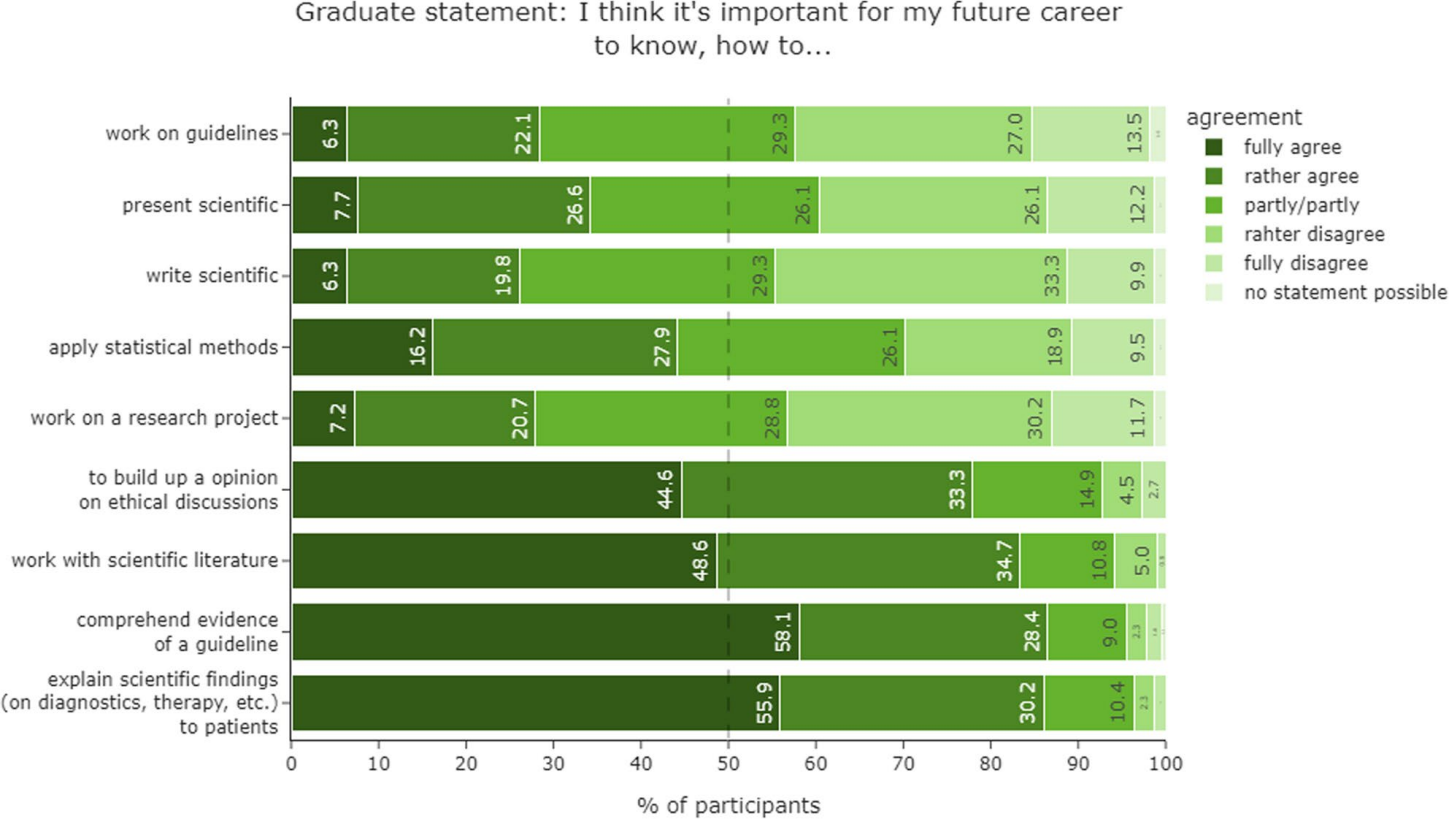
Relevance of different topics in later professional life. Relevance assessment of students that are about to graduate (> 6th AY) for scientific skills in later professional life (*n* = 222)

In our survey we included a relevance assessment of scientific skills for future professional practice (*n* = 3005). Students assigned comparatively low relevance to scientific activities that do not take place in the direct patient environment. For example, less than 27% thought that they will work on a research project or write scientific texts in their future career. In contrast, nationwide most students strongly believed (**~**80% fully or rather agreed) that they will later on have to work with scientific literature, have to understand the evidence of a guideline, have to explain scientific findings to patients as well as have to take a professional position on ethical issues (Fig. 4). Nationwide across all AYs, most students stated that they would like to receive more scientific training (62.2%, data not shown) and the majority would like to have a compulsory scientific course (71.3%).

The desire for a compulsory science curriculum and the high relevance of the general patient-related scientific activity in the later professional activity also reflected in the desired curriculum (Table 3). Dealing with literature and its critical analysis as well as explaining findings to patients was favoured in compulsory courses (seminar or practical). Ethics and good scientific practice were wished for as lectures. Scientific practice as data documentation, statistical analysis, writing and presenting scientific data were primarily requested in optional courses. Students that were about to graduate focused more clearly on scientific literature and its critical analysis in comparison to undergraduates. Less than 6% voted for “no implementation” independent of the topic.

**Table 3:** Preferred curricular implementation formats for competency domains

**Competency domain**	**1-5th AY**				**6th AY**			
	**seminar/practical training (%)**	**lecture (%)**	**elective (%)**	**no imple-mentation (%)**	**seminar/practical training (%)**	**lecture (%)**	**elective (%)**	**no imple-mentation (%)**
Scientific Method	45.3	36.4	41.9	2.2	53.8	39.5	33.2	3.1
Ethics in Science	40.5	48.9	33.4	3.3	42.2	47.5	31.8	4
Good Scientific Practice	30.9	42.1	33.2	4	37.2	45.3	31.8	3.6
Literature Research	41.1	34.2	37.3	3.6	56.5	31.8	28.7	2.7
Critical Literature Analysis	40.5	34.2	36.8	3	55.6	32.7	32.7	0.9
Research Practice	38.7	23.8	47.6	3.3	37.7	24.7	45.7	6.3
Data Documentation and Visualisation	27.5	32.8	42.5	4	30	29.6	39.9	4
Statistical Data Analysis	34	36.3	38.8	3.2	42.6	34.1	40.4	3.1
Scientific Writing	31.5	27.7	47.3	2.8	32.7	26.9	46.2	4
Scientific Presenting	28.8	25.1	49.6	6.1	29.1	27.4	52.5	5.8
Explain scientific findings to patients	51.4	37.5	31.8	5	55.6	35.4	30.9	5.8

The preferred formats as reported by students from the 1st–5th academic year (*n* = 2783) compared to students from the ≥6th academic year (*n* = 222). Values represent the percentage of students endorsing each teaching format (multiple selections possible)

## Discussion

The integration of scientific training in medical education varies, but the adaptation and overhaul of scientific training in medical schools is voiced continuously [25]. This study seeks to analyse the current situation in Germany from a student’s perspective.

### Current state of scientific training in Germany

In our study we reveal a concerning disparity in the perception and evaluation of competencies in scientific training. Only 53.8% of students were aware of their scientific education over all AYs. There is no examination existent in their curriculum stated 60.8% (≥ 6AY). This suggests that despite medical schools reporting obligatory science curricula and assessments [26], these implementations are not effectively communicated to students. The implementation of scientific theory courses (40%) and practical experiences (30.8%) were also reported [22], raising questions about whether students and faculties share a common understanding of scientific education. Better communication between faculties and students seems important to reach a shared view on curricular scientific education. Generally, students feel inadequately trained in scientific skills, with only a minority assessing their qualification at independent level (Fig. 2A). While around half of students beyond > 6 AY feel competent in literature search (52.7%) and scientific writing (44.1%), less feel competent in practical skills like study design (19.9%), research project development (25.7%), or transferring scientific findings without supervision to patients (19.8%). These findings align with other German studies, highlighting deficiencies in skills such as empirics, hypothesis design, and applied statistics [22, 27–30]. Elsewhere, a lack of knowledge in practical applications, such as unsupervised study planning and conduct, was reported [31]. Insufficient practical training and opportunities to participate in research projects are major concerns [32, 33], which is supported by our analysis by low self-assessment scores and overall low competence test results (Fig. 2B). Especially questions on empirics and practical applications of science were less often answered correctly. These deficits extend to residency level, questioning the preparedness of young physicians in applying basic scientific skills [34]. Moreover, there is a substantial portion of students that state that they “can do nothing”, which is in line with our comprehensive analysis focusing on one German medical school [24]. Particularly with the rise of artificial intelligence, such gaps might be masked by AI assisted processes, potentially further restricting scientific skill development and increasing the risk of insufficient rigorous scientific practice. Overall, the deficiencies found by our analyses call for urgent reforms to foster students’ awareness of science curricula and to improve their scientific education especially in practical matters. Furthermore, success can’t be guaranteed only by exams. Research skills should be trained hands on and during practical tasks in a formative way including feedback several times over the years of study. It could be implemented by authentic assessments that are difficult to outsource completely to artificial intelligence, e.g. data interpretation from bespoke datasets, iterative project work with supervisor feedback, oral discussions of research decisions and limitations. So, a hybrid model can retain summative certainty excluding artificial intelligence (high-stake-exams orally or with MC-questions) and formative authenticity where appropriate usage of artificial intelligence could also be part of the learning objectives (project work/portfolios).

### Factors influencing scientific outcomes measured by self-assessment and competency test

Several key factors enhance scientific competencies. Previous healthcare-related studies provide an advantage due to prior exposure to scientific concepts and practical training (Table 2) The progression through medical school enhances self- and objective-assessment scores, with a noticeable improvement as students approach later AYs (Table 2). Both scores were positively influenced if scientific education was included explicitly in the curriculum, no matter the format. Hren et al. also showed that participation in a scientific course increased students’ attitudes towards science and their self-assessed scientific skill [35]. The significant impact of engaging in research during the medical thesis highlights the value of in-depth practical experiences. Students performing a medical doctoral degree assessed their scientific and clinical skills as more competent compared to non-doctoral students [17]. In line with our findings, Schneider et al. [36] demonstrated that students who are not interested in a medical thesis rated scientific skills significantly less relevant and their competencies as less developed compared to doctoral students. The influence of practical research experiences on the perceived importance of scientific practice has been observed in general [37]. Students who performed research had higher scores in the scientific skill assessment compared to those without research experience [38]. Participation in research led students to rate research as more important and conduct research in their future career [39] and students who performed research have a more positive attitude towards science [40]. Given that a medical thesis is not mandatory in Germany and about 20–30% of students do not engage in it, there is a need to improve scientific education and offer sufficient practical training opportunities for all students, regardless of their intrinsic motivation, to prepare them for life-long learning and a positive attitude towards science. Critical thinking and acting in a clinical context and with patients is essential for every physician.

### Student perspectives and preferences

German students show a clear interest in science and value scientific education, aligning with international studies [31, 32, 41–43]. However, around 75% are dissatisfied with their current science education (Fig. 3). This dissatisfaction is also seen in other countries like Croatia, Canada, and the UK [35, 39–42, 44–46].

Faculties need to translate the fundamentally positive student attitude into mandatory curricular offers, emphasizing the relevance of scientific skills for clinical practice and shared decision-making [47]. Graduate profiles like CanMeds [48] and the German NKLM [21], which advocate for roles such as the ‘Scholar’ or ‘Innovator’, serve as a fundamental basis. But evidence-based clinical decision-making did not improve over AYs and was not positively influenced by practical training during a medical thesis [49]. Therefore, clinical decision making by evidence and the analyses of epidemiological data should also become more present when planning and implementing scientific education in medical curricula. Students also voice concerns about extending already overloaded curricula [18]. Since time is a significant barrier for research participation [40, 41, 50], increasing curricular content needs careful evaluation. Programs focusing on students interested in research have proven effective [51, 52]. Weaver et al. found that engaging in research during medical school, whether mandatory or voluntary, supports future scientific careers and research conduct [53].

### Limitations and further research

This study is limited by its scope - only 3% of medical students in Germany participated, which is quite like Ratte et al. [18]. Their responses may not fully capture the diversity of experiences and educational environments. In addition, the voucher raffle might have led to incentivization of a special student population. Furthermore, participation by some universities is low, limiting further subgroup analyses and inferences about the local scientific training.

## Conclusion

In conclusion, while the need for enhanced scientific training in medical education is widely acknowledged, significant gaps remain in its implementation in Germany. The incorporation of scientific practical training opportunities in the mandatory curriculum needs to be evaluated and supplemented by focused resources. Students’ perceptions and opinions need to be included in the further process of curricular refinement. This study highlights the urgent need for educational reforms that are transparent, accessible, and inclusive of practical scientific training to prepare future physicians for the challenges of modern medical practice and research.

### Practice points

- Scientific education should be fostered in the mandatory curriculum of medical students.
- Scientific education should be integrated in all subjects and longitudinally throughout medical school.
- To support clinical practice, the focus should be on scientific competencies needed for clinical duties.

## Supplementary Information

Supplementary Material 1

Supplementary Material 2

Supplementary Material 3

Supplementary Material 4

Supplementary Material 5

Supplementary Material 6

Supplementary Material 7

## Data Availability

All data generated or analysed during this study are included in the supplement.

## Abbreviations

AY: Academic Year
Fig: Figure
GPA: Grade Point Average
MD: Medicinæ Doctor/Doctor of Medicine
NKLM: Nationaler Kompetenzbasierter Lernzielkatalog Medizin
PhD: Doctor of Philosophy
SD: Standard deviation
Suppl: Supplement
TU Dresden: Technical University of Dresden
