# Supplementary figures and images for: Scientific education in German medical schools: nationwide cross-sectional study reveals student needs and gaps
Source: BMC Med Educ. 2026 May 2;26:729. doi: 10.1186/s12909-026-09311-7 (PMC13151329; doi:10.1186/s12909-026-09311-7)

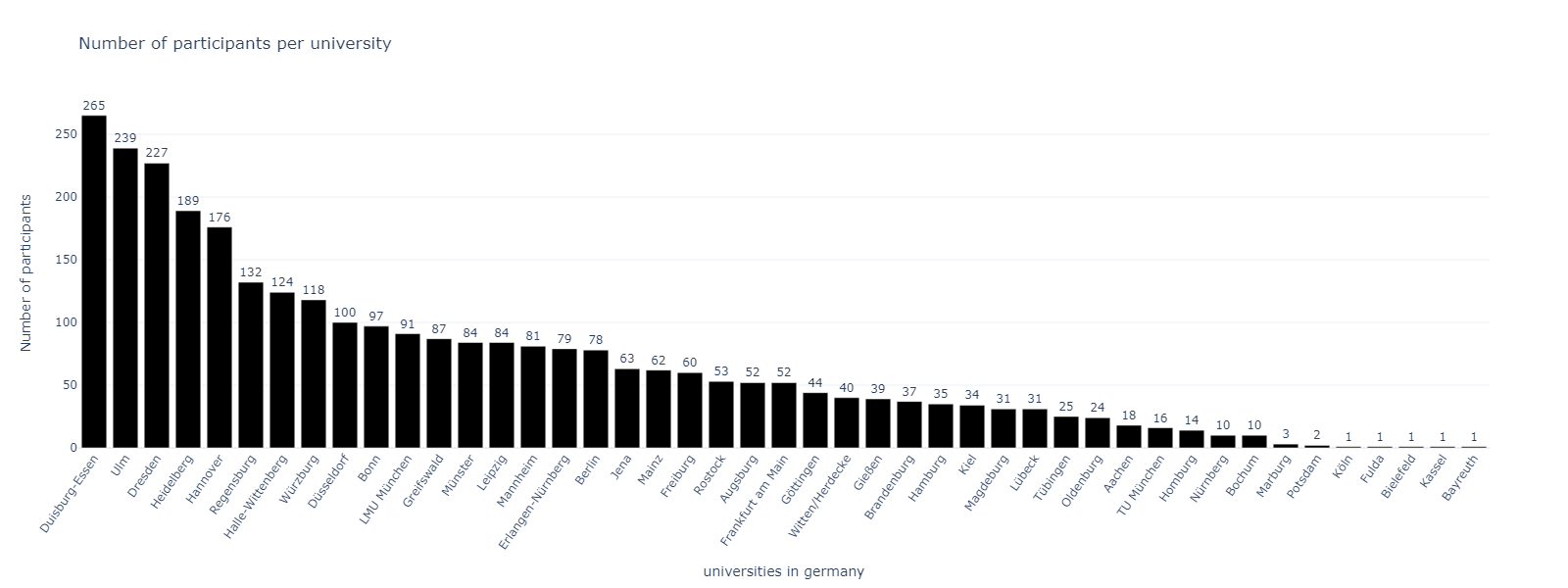


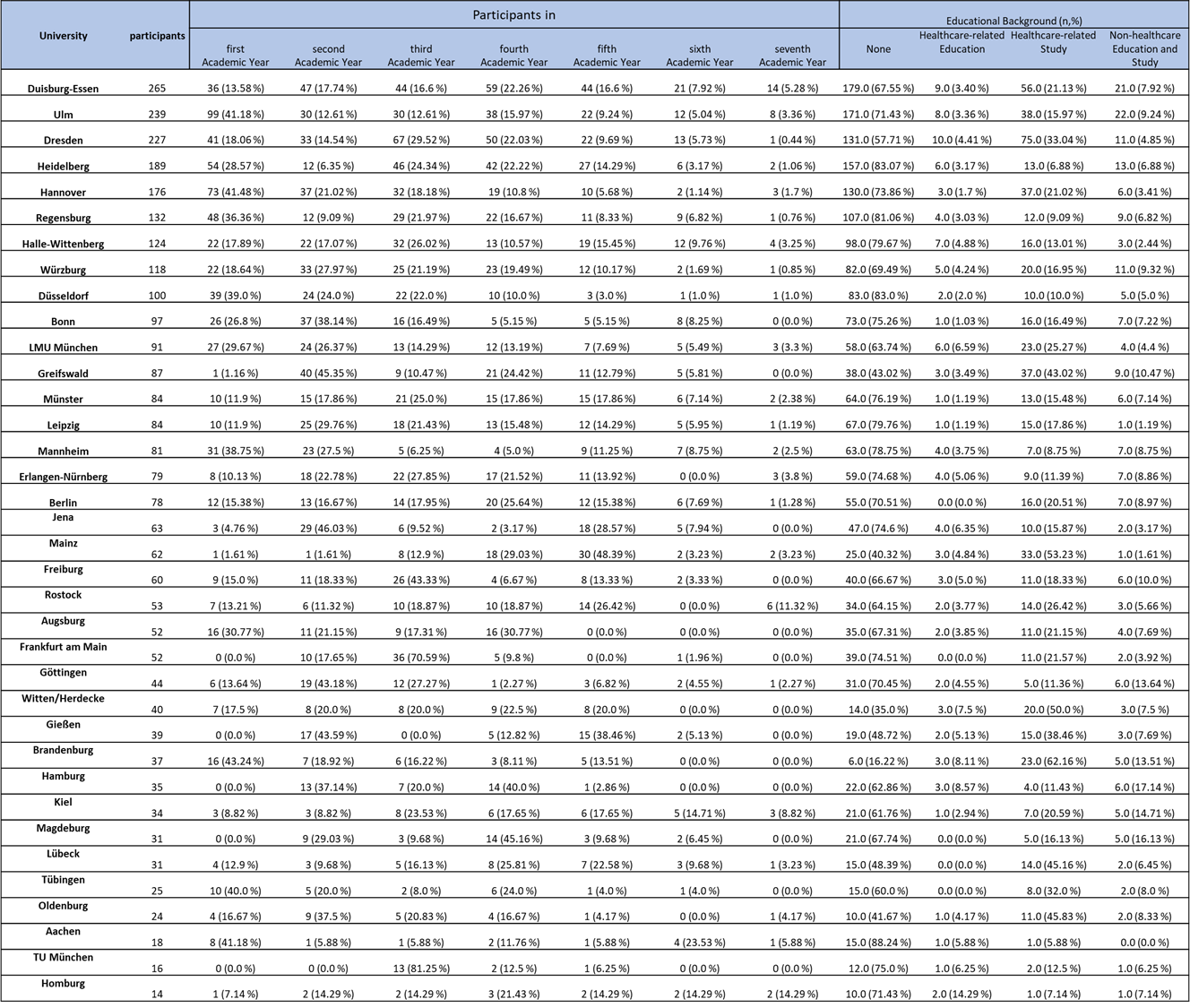

Supplement: Supplementary file 4 — Supplementary Material 4 [file 12909_2026_9311_MOESM4_ESM.docx]

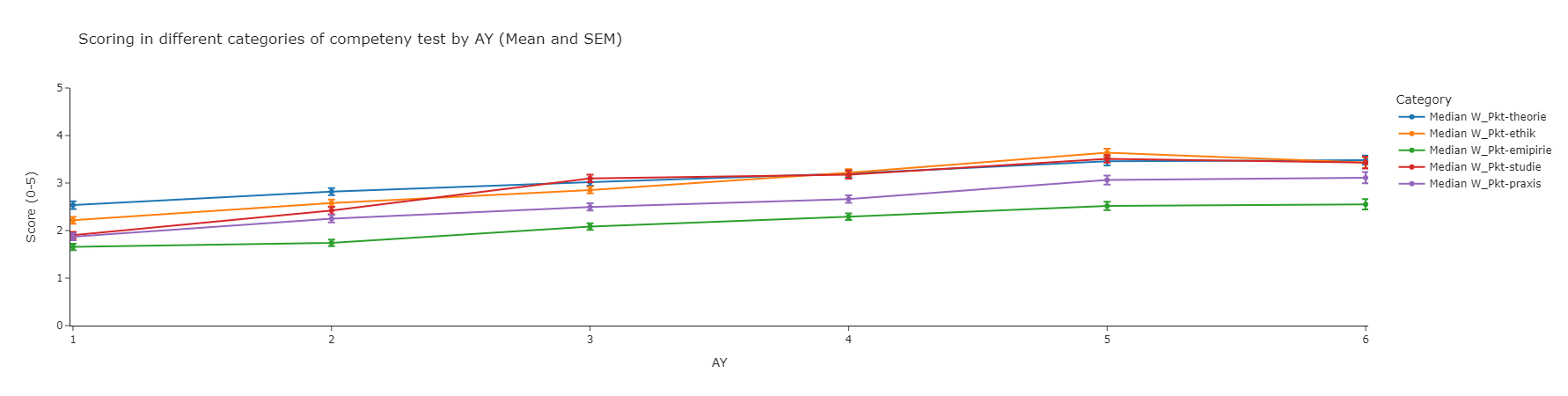

Supplement: Supplementary file 6 — Supplementary Material 6 [file 12909_2026_9311_MOESM6_ESM.png]

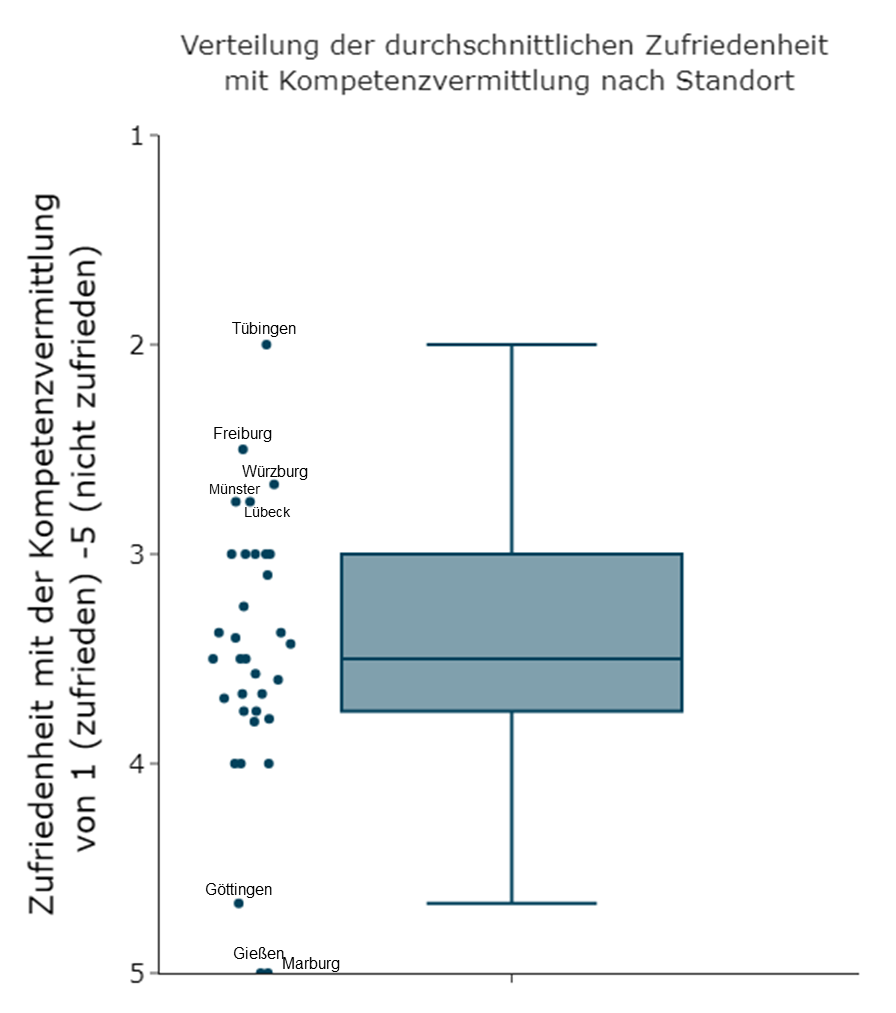

Supplement: Supplementary file 7 — Supplementary Material 7 [file 12909_2026_9311_MOESM7_ESM.png]
