# Supplementary material for: Scientific education in German medical schools: nationwide cross-sectional study reveals student needs and gaps
Source: BMC Med Educ. 2026 May 2;26:729. doi: 10.1186/s12909-026-09311-7 (PMC13151329; doi:10.1186/s12909-026-09311-7)
Supplement: Supplementary file 5 — Supplementary Material 5 [file 12909_2026_9311_MOESM5_ESM.docx]

**
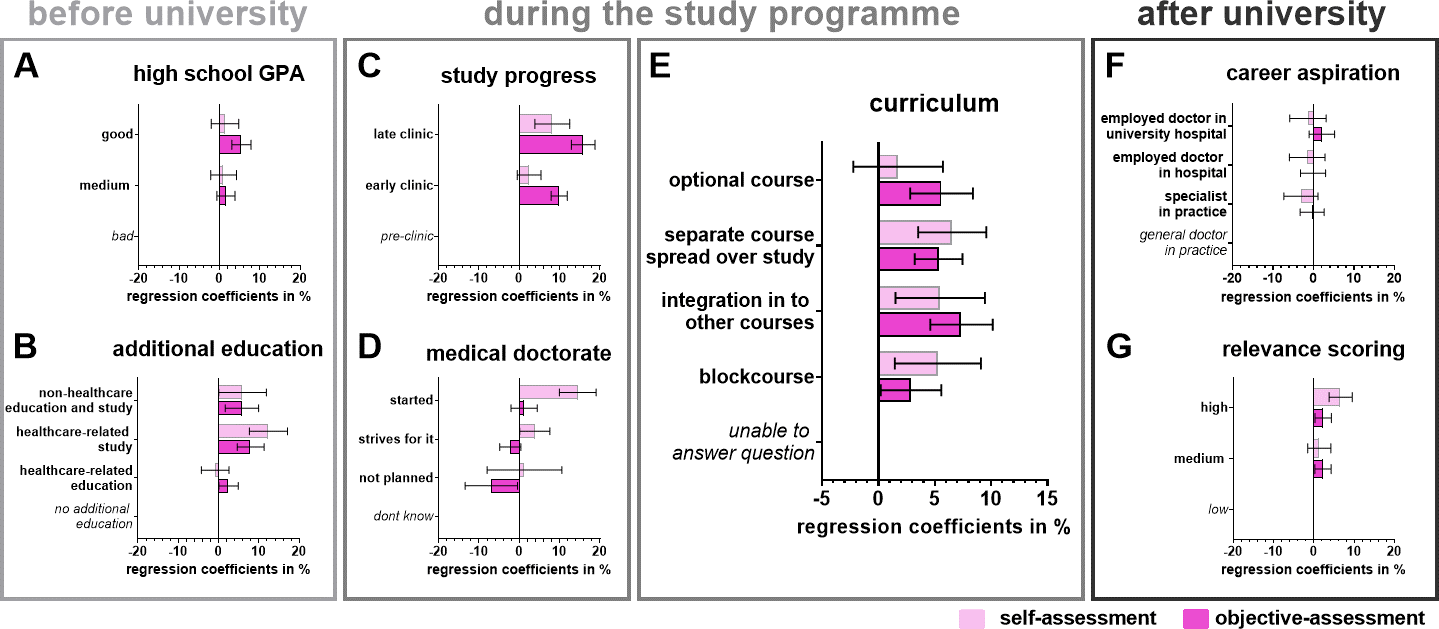
**

**Supplement 5 | Modelling different influences on self-assessed and objective outcomes**

Regression coefficients (in % of assessment score) for the two dependent variables self-assessment (light pink) and objective assessment (dark pink) were obtained from the linear model-analysis (n = 1194) and presented as bar plots with error bars where a normalization has been done to lowest category visualized. The pre-university independent variables, like A) high school GPA or B) additional education are shown on the left. Independent variables during study programmes are shown in the middle, like C) study progress split in 3 phases as pre-clinic: 1-2 AY, early-clinic: 3-4 AY, late clinic: 5-6 AY, D) the doctoral status and E) the science curriculum. On the right side are after universal independent variables like F) career aspirations and G) the relevance scoring for scientific education and their usage in later work.
