# Supplementary material for: Scientific education in German medical schools: nationwide cross-sectional study reveals student needs and gaps
Source: BMC Med Educ. 2026 May 2;26:729. doi: 10.1186/s12909-026-09311-7 (PMC13151329; doi:10.1186/s12909-026-09311-7)
Supplement: Supplementary file 1 — Supplementary Material 1 [file 12909_2026_9311_MOESM1_ESM.pdf]

Survey on scientific competence among medical students in Germany

Dear students,

Thank you for taking the time to complete our survey on scientific work.

During the course of our studies and professional life, we all come into contact with scientific literature or will work scientifically - whether through a doctoral degree, a guideline or simply out of interest. Scientific competence is therefore an important educational goal in medical school!

With our survey, we would like to ascertain your assessments and existing knowledge on the topic of scientific competence and ask you what you would like or would have wished for in terms of teaching the basics of scientific work during your studies. We are surveying all medical students in Germany.

The survey will take about 15 minutes of your time. Your answers will be kept anonymous and strictly confidential. The goal is to use the results to further build a longitudinal science literacy curriculum according to your needs and to prepare you in the best possible way for your later professional life and, if applicable, for your doctoral degree. Thank you very much for your support!

Student Research Group of the Medical Faculty Dresden
(Students Nadja Jahn, Maximilian Vogt, Enrik Geißler, Rona Geißler, Jean-Paul Bereuter and teaching coordinator Dr. rer. nat. Lydia Günther)

There are 52 questions in this survey.

Personal data

How old are you? *

Please choose **only one** of the following:

- 17
- 18
- 19
- 20
- 21
- 22
- 23
- 24
- 25
- 26
- 27
- 28
- 29
- 30
- 31
- 32
- 33
- 34
- 35
- 36
- 37
- 38
- 39
- 40
- 41
- 42
- 43
- 44
- >45
- Other

in years

Which gender would you assign yourself to? *

Please choose **only one** of the following:

- male
- female
- diverse
- no specification

What grade did you graduate from high school with? *

Please choose **only one** of the following:

- 0.7
- 0.8
- 0.9
- 1.0
- 1.1
- 1.2
- 1.3
- 1.4
- 1.5
- 1.6
- 1.7
- 1.8
- 1.9
- 2.0
- 2.1
- 2.2
- 2.3
- 2.4
- 2.5
- 2.6
- 2.7
- 2.8
- 2.9
- 3.0
- 3.1
- 3.2
- 3.3
- 3.4
- 3.5
- 3.6
- 3.7
- 3.8
- 3.9
- 4.0
- no specification
- Other

Have you already started or completed an apprenticeship / studies before medical school? *

Please choose **only one** of the following:

- yes
- no

All apprenticeships / studies that were pursued for more than 1 semester count.

What studies/apprenticeship have you completed? *

Only answer this question if the following conditions are met:
Answer was 'yes' at question '4 [Vorbildung]' (Have you already started or completed an apprenticeship / studies before medical school?)

Please write your answer here:

How long have you been doing your study/apprenticeship? *

Only answer this question if the following conditions are met:
Answer was 'yes' at question '4 [Vorbildung]' (Have you already started or completed an apprenticeship / studies before medical school?)

Please choose **only one** of the following:

- <1
- 1
- 2
- 3
- 4
- 5
- 6
- 7
- 8
- 9
- 10
- >10
- Other

data in years

Where are you studying at the moment? *

Choose one of the following answers
Please choose **only one** of the following:

- Aachen
- Augsburg
- Berlin
- Bielefeld
- Bochum
- Bonn
- Brandenburg
- Dresden
- Duisburg-Essen
- Düsseldorf
- Erlangen-Nürnberg
- Frankfurt am Main
- Freiburg
- Gießen
- Göttingen
- Greifswald
- Halle-Wittenberg
- Hamburg
- Hannover
- Heidelberg
- Homburg
- Jena
- Kassel
- Kiel
- Köln
- Leipzig
- Lübeck
- Magdeburg
- Mainz
- Mannheim
- Marburg
- LMU München
- TU München
- Münster
- Nürnberg
- Oldenburg
- Regensburg
- Rostock
- Tübingen
- Ulm
- Witten/Herdecke
- Würzburg
- Other

Which form of study are you doing?
*

Choose one of the following answers
Please choose **only one** of the following:

- regular studies
- model studies
- I do not know for sure
- Other

You can orientate yourself on the type of the 1st state examination at your university! (Physikum = regular course of study, equivalent = model course of study)

What semester are you currently in? *

Please choose **only one** of the following:

- 1. semester
- 2. semester
- 3. semester
- 4. semester
- 5. semester
- 6. semester
- 7. semester
- 8. semester
- 9. semester
- 10. semester
- 11. semester
- 12. semester
- 13. semester
- >13. semester
- not associated with a semester

Neither previous courses of study nor semesters on leave/research semesters count. If you are currently on leave of absence, tick the last completed semester.

With what grade did you pass your preliminary medical examination (Physikum)?

Only answer this question if the following conditions are met:
Answer was 'not associated with a semester' *or* '12. semester' *or* '11. semester' *or* '10. semester' *or* '6. semester' *or* '>13. semester' *or* '7. semester' *or* '8. semester' *or* '9. semester' *or* '5. semester' *or* '13. semester ' at question '9 [FS]' (What semester are you currently in?) *and* Answer was 'I do not know for sure' *or* 'regular studies' at question '8 [Studienform]' (Which form of study are you doing?)

Please choose **only one** of the following:

- 1,0
- 1,5
- 2,0
- 2,5
- 3,0
- 3,5
- 4,0
- no specification
- Other

With what grade did you pass your equivalent to the preliminary medical examination (Physikum)?

Only answer this question if the following conditions are met:
Answer was '9. semester' *or* '13. semester ' *or* '12. semester' *or* '11. semester' *or* '10. semester' *or* '>13. semester' *or* 'not associated with a semester' *or* '5. semester' *or* '6. semester' *or* '7. semester' *or* '8. semester' at question '9 [FS]' (What semester are you currently in?) *and* Answer was 'model studies' at question '8 [Studienform]' (Which form of study are you doing?)

Choose one of the following answers
Please choose **only one** of the following:

- 1,0
- 1,5
- 2,0
- 2,5
- 3,0
- 3,5
- 4,0
- no specification
- Other

After completing your specialist training, which of the following activities are you most likely to consider? *

Please choose **only one** of the following:

- employed doctor at a **university hospital**
- employed doctor at a **hospital**
- specialist **in own doctors office or MVZ (Medical care center)** (except general medicine)
- **general practitioner** in own doctors office or MVZ (Medical care center)
- employed doctor at a **research institute (not at a university hospital)**
- **employed doctor in the public health service or at a health insurance company**
- **employed doctor in the industry**
- **Other**

About the doctoral degree

What stage is your doctoral degree at? *

Please choose only one of the following:

- **I am aiming for it.**
- **I have started it.**
- **I have completed it.**
- **I cancelled it.**
- **I am still undecided.**
- **I don't want to do a doctoral degree.**
- **Other**

In which field are you doing / have you done your doctoral degree?
*

Only answer this question if the following conditions are met:
Answer was 'I cancelled it.' *or* 'I have completed it.' *or* 'I have started it.' at question '13 [PromoStand]' (What stage is your doctoral degree at?)

Please choose only one of the following:

- **clinical / conservative speciality**
- **clinical / surgical speciality**
- **experimental / theoretical speciality**
- **Other**

In the following, the doctoral degree is divided into three areas: according to clinically conservative, surgical speciality and basic research

How would you rate the following statements?
*

Only answer this question if the following conditions are met:
Answer was 'I cancelled it.' *or* 'I have completed it.' *or* 'I have started it.' at question '13 [PromoStand]' (What stage is your doctoral degree at?)

Please choose the appropriate response for each item:

	fully agree	tend to agree	partly/partly	tend to not agree	do not agree at all	no statement possible
I am satisfied with the results of my doctoral degree.	○	○	○	○	○	○
I am satisfied with the supervision of my doctoral degree.	○	○	○	○	○	○
I am satisfied with the topic of my doctoral degree.	○	○	○	○	○	○

How do you evaluate the following statements?
Completing a doctoral degree has a big impact...... *

Please choose the appropriate response for each item:

	fully agree	tend to agree	partly/partly	tend to not agree	do not agree at all	no statement possible
on the quality of patient care	○	○	○	○	○	○
on the career	○	○	○	○	○	○
on the salary	○	○	○	○	○	○
on the social reputation	○	○	○	○	○	○

Are you completing your doctoral degree as part of a graduate/doctoral program? *

Only answer this question if the following conditions are met:
Answer was 'I cancelled it.' *or* 'I have completed it.' *or* 'I have started it.' at question '13 [PromoStand]' (What stage is your doctoral degree at?)

Please choose only one of the following:

- **yes**
- **no**
- **no specification**

Science curriculum at your university

Does your university have a **mandatory curriculum on scientific competences** for all students? *

Please choose only one of the following:

- **yes**
- **no**
- **no statement possible**

How is the scientific curriculum currently organised? *

Only answer this question if the following conditions are met:
Answer was 'no' at question '18 [Pflichtcurr]' (Does your university have a mandatory curriculum on scientific competences for all students?)

Please choose only one of the following:

- **as a preliminary optional subject**
- **as a clinical elective subject**
- **as a facultative course**
- **no statement possible**
- **Other**

How is the mandatory curriculum organised? *

Only answer this question if the following conditions are met:
Answer was 'yes' at question '18 [Pflichtcurr]' (Does your university have a mandatory curriculum on scientific competences for all students?)

Please choose only one of the following:

- **as a block course**
- **as a separate course spread over the study**
- **integrated into other courses and distributed throughout the study**
- **no statement possible**
- **Other**

Is there a practical course/project on scientific work at your university? *

Please choose only one of the following:

- **yes**
- **no**
- **no statement possible**

(Perspective for every student according to the draft bill for the new "Approbationsordnung")

How long does this practical course last? *

Only answer this question if the following conditions are met:
Answer was 'yes' at question '21 [PrakWiss]' (Is there a practical course/project on scientific work at your university?)

Please choose only one of the following:

- **1 week**
- **2 weeks**
- **3 weeks**
- **4 weeks**
- **5 weeks**
- **6 weeks**
- **7 weeks**
- **8 weeks**
- **9 weeks**
- **10 weeks**
- **11 weeks**
- **12 weeks**
- **>12 weeks**
- **no statement possible**

Are scientific competencies already specifically tested in an exam? *

Please choose only one of the following:

- **yes**
- **no**
- **no statement possible**

facultative competence test on scientific work

Below you will have the opportunity to test your scientific skills with 25 multiple-choice questions. Many of the contents will be tested during the course of your study or in the state examinations. You will apply them in practical scientific work. At the end of the survey you can see your score directly. This can show you where you stand professionally!

These questions are facultative and it is possible to continue without these by clicking "Next" below.

The final submission at the end of the questionnaire is important because it is the only way your answers will be included in the evaluation. Please support our student project!

How long does the German Research Foundation (Deutsche Forschungsgesellschaft, DFG) recommend in its guidelines to retain raw data to ensure good scientific practice?

Please choose only one of the following:

- **5 years**
- **10 years**
- **15 years**
- **20 years**
- **25 years**

Which statement about ensuring good scientific practice is most likely to be correct?

Please choose only one of the following:

- **Original data are subject to confidentiality and are the property of the group.**
- **The group leader is the last author of a publication.**
- **All authors agree to the final version of a manuscript to be published.**
- **The first author of a publication bears the legal responsibility for the content due to his/her prominent position.**
- **The Dean of Research of a faculty must approve a publication (right of veto).**

For your doctoral project it may be necessary for you to gain knowledge in dealing with animals and animal experiments. The 3R rule is explained in an animal testing course. It is understood as

Please choose only one of the following:

- **the sequence for stress experiments - Rest, Resist, Rest.**
- **the 3 standard test animals - Rat, Rabbit, Raccoon.**
- **the categorisation of animal testing laboratories into safety zones - radioactive zone, residual zone, recovery zone.**
- **the principle - Replace, Reduce, Refine**
- **the principle of designing safe experiments with drugs - right animal, right application, right dosage**

Which statement about working with animals in research is correct?

Please choose only one of the following:

- **Once one has obtained the licence to practice medicine, one is authorized to perform animal experiments.**
- **Animal experiments are always permitted as long as they are well justified and a case number plan is available.**
- **The conditions under which the animals are kept are determined by the head of the experiment.**
- **Anyone who performs authorised animal experiments is obliged to report the animals used to the responsible authority.**
- **Animal testing projects authorised by the authorities run for an unlimited period of time.**

You would like to search for all publications on the topic of "ACE-2 inhibitors" in the biomedical meta-database PubMed in which an interaction with antipsychotics has been investigated. Which search term is most suitable for this?

Please choose only one of the following:

- **"Antipsychotic Agents"[Mesh] AND "Angiotensin-Converting Enzyme Inhibitors"[Mesh]**
- **"Antipsychotic Agents"[Mesh] OR "Angiotensin-Converting Enzyme Inhibitors"[Mesh]**
- **"Antipsychotic Agents" NOR "Angiotensin-Converting Enzyme Inhibitors"[Mesh]**

○ "Antipsychotic Agents" AND "Angiotensin-Converting Enzyme Inhibitors"
○ none of the named

The ethical principles of medical research on humans were established

Please choose only one of the following:

○ in the Declaration of Helsinki
○ in the Oslo Accord
○ in the Geneva Pledge
○ in the Roman treaties
○ in the Basic Law for the Federal Republic of Germany

Which statement about scientific ethical principles and the work of ethics committees is most accurate?

Please choose only one of the following:

○ Findings that are obtained in an unethical manner, but which bring significant medical progress, must be researched further.
○ An important principle of medical ethics is the disclosure of all test subjects.
○ The constitutionally guaranteed freedom of science legitimises medical research on humans.
○ Ethics committees have been set up to conduct case number planning for research projects.
○ Ethics committees can have different compositions and also include non-professionals.

The PICO scheme can be used to formulate precise scientific questions.What does it mean?

Please choose only one of the following:

○ Patient, International, Control, Observation
○ Patient, Intervention, Comparison, Outcome
○ Practical, Intervention, Control, Outcome
○ Probe, International, Case, Observation
○ Prospective, Intervention, Case, Outcome

Which statement about the deductive research approach is most accurate?

Please choose only one of the following:

○ There is little prior knowledge of the research topic at the start of the research.
○ Deductive research is less focussed and method-oriented.
○ Conclusions are drawn from a model/theory to the individual case.
○ Deduction stands for the process of theorising.
○ The approach is based on the epistemological approach of empiricism.

What is best understood by a theory?

Please choose only one of the following:

○ It is a description of reality that corresponds to the truth.
○ It is based on a loose collection of theses.
○ It corresponds to a medical guideline and is drawn up by scientists.
○ It provides explanations for each individual case and each phenomenon located within it.
○ It is a structure of hypotheses or laws using defined terms.

What is most likely to represent science?

Please choose only one of the following:

○ It is the totality of well-founded knowledge about a subject area.
○ One of its main characteristics is its well-founded subjectivity.
○ It formulates unsystematic statements about reality.
○ Scientific findings are true and reflect reality.
○ It differs from parasciences in that it is interdisciplinary.

Which statement about peer review in the quality management process of publishers is most accurate? It is a quality control

Please choose only one of the following:

○ by non-professionals from pre-print publications on web-based servers
○ by other scientists prior to publication
○ by the ethics committee of the editor/publisher prior to publication
○ by the research committee of an institution before submission of the manuscript
○ by the Science Council after the publication

The sex of the proband is an example of a characteristic of which scale type?

Please choose only one of the following:

○ nominal scale
○ ratio scale
○ interval scale
○ ordinal scale
○ zero scale

In order to assess further development and risk factors for a rare disease, which of the following types of studies should be prioritized given a small budget and low staffing capacity?

Please choose only one of the following:

○ randomised controlled prospective trial
○ prospective cross-sectional trial
○ prospective intervention trial
○ case-control trial
○ cohort study

In a study on prostate cancer prevention, it is stated that out of 1000 probands with an abnormal PSA value, only around 330 probands actually have prostate cancer. On the other hand, out of 1,000 probands with a negative PSA test result, around 250 probands have prostate cancer. Which key figures/statistical measures are addressed in these lines?

Please choose only one of the following:

○ prevalence and Number Needed To Treat
○ negative and positive predictive value
○ absolute and relative risk reduction
○ Odds ratio and relative risk
○ relative and absolute number of cases

Interventional studies are divided into stages. Which stage applies when a "large study for precise information about effectiveness, tolerability and safety before market approval" is addressed?

Please choose only one of the following:

○ Phase I
○ Phase II
○ Phase III
○ Phase IV
○ Phase V

You would like to investigate the influence of the sex on the tendency to thrombosis after TKA ((total endoprosthesis)) and its hormone-related influence on coagulation. Which statistical test would you most likely use for the data set collected?

Thrombosis after TEP	men	women
yes	450	691
no	481	522

Please choose only one of the following:

○ Chi-squared independence test
○ F-Test
○ Mann-Whitney U test
○ Pearson correlation test
○ Gold standard test

A systematic error in a study that leads to an incorrect estimate of the effect of a medication in terms of the risk of a side effect is called what?

Please choose only one of the following:

○ Simpsons Paradoxon
○ interaction
○ confounder
○ risk factor
○ none of the mentioned

Which statement about habilitation and the awarding of the "venia legendi" status is true in Germany?

Please choose only one of the following:

○ There is a standardised habilitation regulation that applies throughout Germany.
○ The title "venia legendi" is dependent on the acquisition of external funding.
○ Teaching is a basic requirement for the title "venia legendi".
○ It is mandatory to submit 10 publications as a first author.
○ A course on good scientific practice must be attended for the habilitation.

With the help of a statistical significance test, the validity of hypotheses can be checked based on existing data (random sample). Which statement about the p-value is true?

Please choose only one of the following:

○ It indirectly indicates the size of the effect observed on the basis of the available data (e.g. difference between the treatment groups).
○ It indicates how likely it is that the null hypothesis is correct, even though the test produced a significant result (i.e. rejection of the null hypothesis).
○ It indicates the probability of the observed data under the validity of the null hypothesis. In addition to the observed data, the p-value also considers data that are to be regarded as even more extreme (i.e. less probable) with reference to the null hypothesis.
○ It indicates the possibility that the test result is incorrect and the experiment must be repeated.
○ The power of the test (i.e. the probability of detecting a real effect with the test) can be calculated from the p-value, i.e. power = 1 - p-value.

A 50-year-old patient who is very afraid of breast cancer has both breasts examined by her doctor using sonography. Due to an abnormal finding, the patient is referred for mammography. "Dense glandular tissue" is also diagnosed here. The combination of both examinations is estimated to have a sensitivity of 74%. What statement can you most likely make to your patient after both examinations?

Please choose only one of the following:

○ You don't need to worry. Out of 100 women with this finding, 74 are healthy.
○ Regardless of the tests, many other factors play a role in modulating the pre-test probability, such as a family history of breast cancer.
○ The sensitivity of both methods is high enough to confirm the diagnosis of "breast cancer".
○ Out of 1000 people, 10 of whom are ill, the test is false positive in more than 8 cases.
○ Since your patient has no symptoms, the tests must necessarily be wrong.

A total of 20 female test persons were examined for the effect of a blood pressure medication. As part of a randomization, 10 of these were assigned to the control group and 10 to the treatment group. With regard to the predetermined endpoint and the agreed significance level of 5%, no significant difference could be demonstrated. What conclusion can you draw from the results?

Please choose only one of the following:

○ The study should be repeated as the expected effect could not be shown.
○ In order to be able to specify the error probability of overlooking a real effect, it would have been necessary to plan the number of cases in advance.
○ Due to the lack of significance, the study was able to show statistically (with an error of 5%) that the treatment had no (measurable) effect.
○ The non-significant result shows that the number of cases is too small to prove the expected effect.
○ The dose in the treatment group should be doubled in order to show the effect of the treatment more clearly.

Which of the suggested words or abbreviations characterises a citation style frequently used in scientific publications in Germany?

Please choose only one of the following:

○ APA
○ Tunesian
○ HAP
○ SanFrancisco
○ Calibri

A study to prove the efficacy of long-term heparin therapy is being conducted in patients following polytrauma events. The case number planning envisages an n of 100 patients. So far, 70 patients have been acquired. The control group is clearly (statistically significant and relevant) inferior in terms of mortality outcome. Now you come across a publication by a competing international working group that proves the inferiority of the control group. Which statement is correct?

Please choose only one of the following:

○ I still have to randomise the remaining 30 patients and examine them further.
○ It should be considered whether the study will be cancelled.
○ I am free to choose between the options listed under A) and B).
○ The centre for clinical studies must decide on the continuation of the study.
○ The decision lies with the patients, who can decide in which group they want to be treated.

You assume that a certain intervention has an influence on the migration of cells. Using a T-test for independent samples (at a significance level of 0.01), you were unable to demonstrate a significant change in the mean migration index. How do you interpret the following graph?

(N=25 cells/group; bar height = arithmetic mean; empirical standard deviation = error bar)

Please choose only one of the following:

○ The hypothesis of an influence of the intervention on migration could be statistically refuted.
○ The assumed influence of the intervention on migration could not be demonstrated because too few cells were analysed.
○ The postulated influence of the intervention on migration could lead to some cells migrating more and others less. However, statistical proof of this cannot be provided by comparing mean values (T-test).
○ In order to be able to statistically demonstrate the postulated influence of the intervention on migration, a paired T-test should have been used, in which two random cells are always compared with regard to their difference in migration.
○ Although the graph suggests that the intervention increases the heterogeneity of the migration behaviour of the cells, such an effect can be ruled out by the non-significant test result with an error probability of 1%.

Expectations of studies and career path

How do you rate the following statements? I consider it relevant in my later professional life....? *

Please choose the appropriate response for each item:

	fully agree	tend to agree	partly/partly	tend to not agree	do not agree at all	I cannot estimate
to work with scientific literature	○	○	○	○	○	○
to work on a research project	○	○	○	○	○	○
to apply statistical methods	○	○	○	○	○	○
to write scientific papers	○	○	○	○	○	○
to present posters and talks	○	○	○	○	○	○
to work on a guideline	○	○	○	○	○	○
to understand the evidence of a guideline	○	○	○	○	○	○
to communicate scientific findings (on diagnostics, therapy, etc.) to patients	○	○	○	○	○	○
to take a stance on ethical principles in science	○	○	○	○	○	○

How would you rate your current level of knowledge on the following topics?

The depths of competence are based on the NKLM 2.0, with competence depth 1 corresponding to factual knowledge, 2 to action and reasoning knowledge, 3a to action competence with guidance/control, 3b to action competence without guidance. *

Please choose the appropriate response for each item:

	0 - can't do anything yet	1 - know and name (what)	2 - explain and elaborate (how and why)	3a - perform under guidance	3b - perform independently
research questions and hypotheses	○	○	○	○	○
literature research	○	○	○	○	○
critical literature analysis	○	○	○	○	○
data documentation and visualisation	○	○	○	○	○
statistical data analysis	○	○	○	○	○
scientific writing	○	○	○	○	○
scientific presentations	○	○	○	○	○
ethical principles in science	○	○	○	○	○
rules of "good scientific practice"	○	○	○	○	○
study design	○	○	○	○	○
scientific work/research projects	○	○	○	○	○
communicate scientific findings to patients	○	○	○	○	○

The depths of competence are based on the NKLM 2.0, with competence depth 1 corresponding to factual knowledge, 2 to action and reasoning knowledge, 3a to action competence with guidance/control, 3b to action competence without guidance.

Which topics for acquiring scientific competences should be anchored in the curriculum and in what form? (multiple answers possible) *

	obligatory teaching format (seminars/practical courses)	lectures	compulsory course for the doctoral degree	elective subjectoffer	do not anchor	no statement possible
scientific method (as a research process)	☐	☐	☐	☐	☐	☐
research practice (practical skills)	☐	☐	☐	☐	☐	☐
literature research	☐	☐	☐	☐	☐	☐
critical literature analysis	☐	☐	☐	☐	☐	☐
data documentation & visualisation	☐	☐	☐	☐	☐	☐
statistical data analysis	☐	☐	☐	☐	☐	☐
scientific writing	☐	☐	☐	☐	☐	☐
scientific presentation	☐	☐	☐	☐	☐	☐
ethics in science	☐	☐	☐	☐	☐	☐
"good scientific practice"	☐	☐	☐	☐	☐	☐
study design	☐	☐	☐	☐	☐	☐
communicate scientific findings to patients	☐	☐	☐	☐	☐	☐

Multiple selections possible

How would you rate the following statements? *

Please choose the appropriate response for each item:

	fully agree	tend to agree	partly/partly	tend to not agree	do not agree at all	no statement possible
I would like to be taught scientific work in the compulsory curriculum.	○	○	○	○	○	○
I am satisfied with the teaching of scientific skills at my faculty.	○	○	○	○	○	○
I would generally like to be taught more skills for scientific work.	○	○	○	○	○	○
The COVID-19 pandemic has increased the relevance of scientific skills for medical students.	○	○	○	○	○	○
I am satisfied with the communication of local rules and regulations for the doctoral degree.	○	○	○	○	○	○

Thank you for completing our survey on scientific comeptence.

If you have any questions or comments, please write to lcwiss@tu-dresden.de.

We appreciate your feedback.

The competition for 15 Medimeisterschaften tickets and Amazon vouchers has already ended. The winners have already been contacted by email.

05-25-2023 – 21:33

Submit your survey.
Thank you for completing this survey.
